# Caregiver burden in informal caregivers of cardiac patients: a mixed-methods systematic review

**DOI:** 10.1186/s40359-026-03973-0

**Published:** 2026-02-05

**Authors:** Fateme Beheshtaeen, Zahra Molazem, Majid Najafi Kalyani, Zinat Mohebbi, Reza Moshfeghinia, Mohammad Goudarzi Rad

**Affiliations:** 1 Department of Nursing, School of Nursing and Midwifery, Shiraz University of Medical Sciences, Shiraz, Iran; 2 Research Center for Psychiatry and Behavioral Sciences, Shiraz University of Medical Sciences, Shiraz, Iran; 3 Lawrence S. Bloomberg Faculty of Nursing, University of Toronto, Toronto, Canada; 4 Peter Munk Cardiac Centre, Toronto, General Hospital, University Health Network, Toronto, Canada

**Keywords:** Informal caregivers, Caregiver burden, Cardiovascular disease, Systematic review

## Abstract

**Background:**

Informal caregivers, frequently family members or close acquaintances, offer uncompensated care and support to individuals with cardiovascular diseases, confronting diverse challenges such as mental stress, financial strain, and the overall burden of care. Addressing these challenges is vital for both caregivers and patients. This systematic review seeks to identify the factors contributing to caregiver burden among informal caregivers of cardiovascular patients.

**Methods:**

PubMed, Scopus, ProQuest, Web of Science, and Science Direct were searched from 2000 until July 2023, following the PRISMA guidelines, to systematically retrieve relevant papers. The review incorporated quantitative, qualitative, and mixed-methods studies.

**Results:**

Forty-two studies were included in the systematic review. Data synthesis adhered to the Joanna Briggs Institute (JBI) methodological guidance using a convergent integrated approach. Five categories emerged: individual characteristics (demographic characteristics of the caregiver and patient-related factors), confronting multifaceted tensions (physical problems, mental health problems, and financial burden), role strain in daily life, inadequate health literacy, and lack of support.

**Conclusion:**

This review underscores that informal caregivers of cardiac patients encounter substantial burdens. These burdens are intensified by challenges, including insufficient knowledge, restricted social support, and suboptimal communication from both family members and healthcare providers, resulting in increased physical and psychological stress. It is crucial to evaluate informal caregivers for these burdens and offer the required support. Clinical implications include routine assessment of caregiver burden, providing tailored educational interventions to enhance health literacy, and offering financial and psychosocial support to mitigate risks specific to cardiovascular disease caregiving.

**Supplementary Information:**

The online version contains supplementary material available at 10.1186/s40359-026-03973-0.

## Introduction

Worldwide, cardiovascular diseases (CVDs) stand as the foremost contributor to morbidity and mortality, imposing significant societal and economic burdens [1]. Based on statistics from the Global Burden of Disease, Injuries, and Risk Factors Study (GBD) 2019, there was a notable increase in the overall population regarding the total count of CVD cases (from 272 million to 523 million), fatalities (from 12.1 million to 18.6 million), and disability-adjusted life years (DALY, from 279.8 million to 393.1 million) between 1990 and 2019 [2]. On a global scale, CVD remains the predominant health challenge. Incidence rates are on an upward trajectory in regions beyond those classified as high-income nations, with instances of a reversal in the declining trend even observed in select high-income locales [2, 3].

The complications of CVD extends far beyond the physical health of patients, profoundly affecting their psychological well-being, financial stability, and the lives of their caregivers [4]. From the moment of diagnosis, patients often grapple with overwhelming anxiety, depression, and a persistent fear of future health complications [5, 6]. Furthermore, the financial strain is substantial, with the cost of medications, medical procedures, and lifestyle modifications adding up quickly [7]. These CVD-specific burdens, such as managing acute events like myocardial infarctions or chronic conditions like heart failure, place unique demands on informal caregivers [8].

Caring for patients with chronic illnesses is a critical aspect of contemporary society [9]. A caregiver is a person who provides direct assistance and support to individuals needing help with daily activities. Informal caregivers, offer unpaid assistance, typically comprising family members, neighbours, or close acquaintances [10].

Informal caregivers play a pivotal role in offering support, enhancing patient compliance, and reducing the burden on healthcare systems [11] but due to chronicity and complexity of CVDs, the management of CVD presents a multifaceted challenge, encompassing lifestyle modifications, medication adherence [12], monitoring the patient’s blood pressure, engaging in exercise and arranging medical appointments [13]. Balancing the needs of patients while maintaining one’s own quality of life can be a Herculean task, illustrating the multifaceted nature of the burden that CVD imposes on both individuals and those who care for them [14, 15].

Liu et al. (2020) conducted a concept analysis defining caregiver burden as “the level of multifaceted strain perceived by the caregiver from caring for a family member and/or loved one over time,” encompassing antecedents (caregiver characteristics and care demands), attributes (physical, psychological, emotional, social, and financial strains), and consequences (health impacts on the caregiver) [14].

Informal caregivers aiding individuals suffering from cardiac disease encounter a myriad of difficulties that can influence both the caregivers themselves and the individuals under their care. These challenges encompass a spectrum of domains, including mental, financial, and the overall burden of care [16]. In the realm of mental well-being, caregivers frequently contend with elevated levels of stress, anxiety, and depression, largely attributable to the demanding nature inherent in caring for a family member afflicted by cardiac diseases [17]. The challenges and requirements faced by informal caregivers are contingent upon both the caregiver’s circumstances and the patient’s condition. The caregiver’s burden is influenced by the caregiver’s background, culture, illness, and the caregiving context [18]. Caregivers of cardiac individuals often feel increased pressure when patients struggle with self-care, leading to psychological distress [19, 20]. The emotional state of the patient significantly affects caregiver well-being; positive interactions can enhance resilience, while negative experiences may cause burnout [21]. Moreover, caregiver can also experience profound rewards, including personal growth, strengthened relationships, and a sense of purpose. Research indicates that recognizing these dual aspects can lead to better support systems for caregivers, fostering resilience and enhancing their overall well-being [22, 23].

Limited research, in the form of systematic reviews, exists regarding factors influencing caregiver burden in patients with cardiac disease. In a study conducted by Bjornnes et al. (2019), burden is portrayed through caregivers’ lived experiences as feelings of insecurity, being overwhelmed by responsibilities, lack of clear and concise discharge information, and inadequate follow-up support during the early recovery period after cardiac surgery [24]. Another study by Suksatan et al. (2022) focused on the relationship between the caregiver burden of informal caregivers of heart failure patients and their health outcomes. In this study, caregiver burden is described as a significant strain linked to caregiving demands in heart failure patients, associated with negative caregiver outcomes including depression, reduced quality of life, and increased healthcare utilization [25].

Although prior systematic reviews have examined caregiver burden in specific cardiac subgroups, there has been no comprehensive mixed-methods synthesis spanning the full spectrum of adult cardiovascular conditions. Therefore, this review addresses that gap by integrating quantitative and qualitative evidence across CVD diagnoses to identify common and condition-specific contributors to caregiver burden. By recognizing existing literature on caregiver burden, this study seeks to clarify its novel contributions, specifically in identifying gaps in current knowledge and developing targeted interventions tailored to the diverse needs of caregivers. This approach not only enhances understanding of caregiver challenges of CVD patients but also positions the study within the broader context of existing research, ultimately aiming to improve support systems that benefit both caregivers and cardiac patients within healthcare frameworks.

## Methods

This study adheres to the guidelines outlined in the Preferred Reporting Items for Systematic Review and Meta-Analysis (PRISMA) (2020) (Table S1) and follows the Joanna Briggs Institute (JBI) methodological guidance for conducting a mixed-methods systematic review in design and reporting [26]. The protocol has been registered with the International Prospective Register of Systematic Reviews (PROSPERO) under registration number CRD42023460294.

### Search strategy

Five electronic databases (PubMed, Scopus, ProQuest, Web of Science, and Science Direct) were searched in the English language from 2000 until July 30, 2023, to ensure comprehensive and up-to-date coverage of the literature. Additionally, the reference lists of included studies were screened for more comprehensive searches. The search strategy, based on PICo (Population: informal caregivers of cardiac patients; Phenomena of Interest: caregiver burden; Context: cardiovascular disease), used key terms including “Caregiver” OR “Carer(s)” OR “Care Giver” OR “Spouse Caregivers” OR “Family Caregivers” OR “Informal Caregivers” AND “Caregiver Burden” OR “Care Burden” OR “Care Giving Burden” OR “Caregiver Burnout” OR “Caregiver Exhaustion” OR “Caregiver Strain” AND “Heart Disease” OR “Cardiac Diseases” OR “Cardiac Disorder(s)” OR “Heart Disorder(s)” OR “Coronary Artery Bypasses” OR “Coronary Artery Bypass Surgery” OR “Bypass, Coronary Artery” OR “Aortocoronary Bypass” OR “Bypass Surgery, Coronary Artery” OR “Coronary Artery Bypass Grafting” OR “Cardiovascular Disease” OR “Cardiac Event” OR “Myocardial Infarctions” OR “Cardiovascular Stroke” OR “Myocardial Infarct(s)” OR “Heart Attack” OR “Coronary Artery Diseases” OR “Left Main Coronary Artery Disease” OR “Left Main Disease” OR “Left Main Coronary Disease” OR “Coronary Arteriosclerosis” OR “Cardiac Failure” OR “Heart Decompensation” OR “Right-Sided Heart Failure” OR “Myocardial Failure” OR “Congestive Heart Failure” OR “Cardiac Dysrhythmia” OR “Cardiac Arrhythmia” OR “Arrhythmia” OR “Heart Grafting” OR “Heart Transplantations” OR “Cardiac Transplantation” OR “Cardiac Surgical Procedure” OR “Heart Surgical Procedure(s)”. (Table S2).

### Inclusion and exclusion criteria

#### Types of studies

##### Inclusion criteria

This study encompasses quantitative (descriptive design), qualitative, and mixed-methods studies, elucidating caregiver burden factors, challenges, and the needs of informal caregivers of cardiac patients.

##### Exclusion criteria

To maintain homogeneity of the purpose of the study, interventional studies, including randomized clinical trials, quasi-experimental designs, pre-experimental investigations were excluded, as the focus was on identifying contributing factors to caregiver burden rather than evaluating intervention efficacy. Case reports and case studies were excluded because they typically focus on individual cases and provide limited generalizable evidence on contributing factors to caregiver burden. In addition, editorial comments, letters to the editor, review studies, and non-full text articles which their authors were not accessible were excluded.

PICo: Participants, Phenomena of interest, and Context for this study includes:

Participants: Informal (unpaid/family) caregivers of adult patients (aged ≥ 18 years) with any cardiovascular disease.

Phenomena of Interest: Factors contributing to caregiver burden, including challenges and needs.

Context: Caregiving in the context of cardiovascular diseases (e.g., heart failure, acute coronary syndrome, cardiac surgery, arrhythmias).

### Quality assessment

According to the 2018 version of the Mixed Methods Appraisal Tool (MMAT), two independent reviewers conducted quality assessments of the studies. The MMAT is specifically designed for evaluating mixed methods studies, encompassing qualitative, quantitative, or a combination of both approaches. The tool incorporates two screening questions tailored for different study types, with five questions allocated for each of the two potential research design categories to gauge research quality. In MMAT version 2018, the practice of calculating an overall score based on individual criterion ratings is discouraged. Instead, it is recommended to present a more detailed breakdown of the ratings for each criterion, offering a nuanced perspective on the quality of the included studies. The exclusion of studies with low methodological quality is generally advised against [27]. (Table S3 in supplementary material)

### Study selection

After conducting an initial search on the mentioned databases and removing duplicates, two reviewers (F.B., Z.M.) independently screened the identified studies by reading titles and abstracts. Subsequently, both reviewers selected eligible articles based on the inclusion and exclusion criteria.

Data extraction was performed independently by two authors using a standardized form developed prior to extraction, which captured study characteristics (authors’ name, country, individuals’ disease, purpose and main finding of the study), and MMAT 2018 quality criteria.

**Fig. 1:**
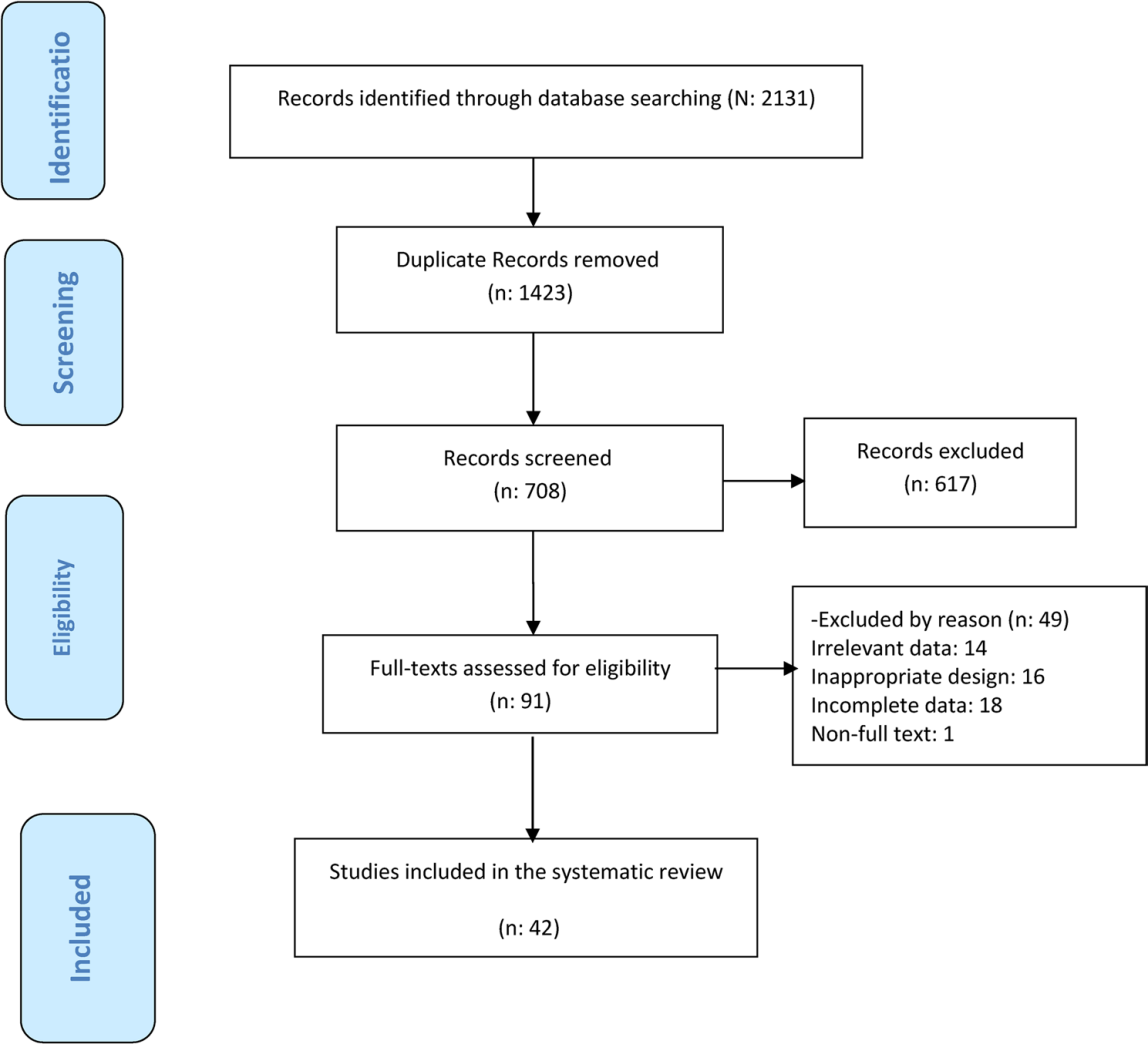
PRISMA diagram for searching resources

In cases where discrepancies or disagreements arose between the two reviewers regarding extracted data, the reviewers first attempted the to resolve these differences through detailed discussion and examination of the original study reports. If consensus was not reached through discussion, a third independent reviewer (M.N.) was consulted to adjudicate the disagreement and make a final determination. Ultimately, 42 eligible studies were incorporated into the systematic review (See Fig. 1).

### Data extraction

The data extracted from each included study comprises the author’s name, publication year, individuals’ disease, sample size, purpose and main finding of the study (Table 1).

**Table 1:** The characteristics of included studies

ID	Authors’ Name/year	Country	Study Design	Individuals’ Disease	Sample Size	Purpose of Study	Main Findings
1	Rosenkilde et al. 2023 [28]	Denmark	Qualitative	OHCA Survivors	N: 25	To explore how family caregivers of OHCA survivors experience the burden.	Caregivers experience unexpected loneliness, fear of loss, and challenges adjusting to new daily routines
2	Thomas et al. 2023 [29]	USA	Longitudinal	LVAD	N: 60	To explore the link between caregiver burden at baseline and patient recovery post long-term LVAD implantation.	Initial high caregiver burden does not correlate with patient recovery in the first year post-LVAD implantation.
3	Laflamme et al. 2022 [13]	Canada	Cross-Sectional	CVD	N: 181	To explore links: attachment, caregiver burden, psychological distress in cardiac context.	Low caregiver burden, yet significant mediating role in caregiver depression, anxious attachment, and avoidant attachment.
4	Yoltay et al. 2022 [30]	Turkey	Cross- Sectional	Open Heart Surgery	N: 44	To assess burden and stress in companions of elderly patients post open-heart surgery.	Mild burden, stress; high burden, increased stress
5	Bohm et al. 2021 [31]	Sweden, Denmark, Italy, the Netherlands and the United Kingdom	Cross-Sectional (Comparative)	OHCA survivors and STEMI	-Control: Caregiver of STEMI N: 108 -Case: Caregiver of OHCA survivors N: 272	To describe caregiver burden and HRQoL in OHCA-survivor caregivers	Similar caregiver burden in OHCA and STEMI; quality of life scores within normative levels in both
6	Cooney et al. 2021 [32]	USA	Prospective	HF	N: 100	To study how changes in social support and patient-caregiver relationships influence the link between HF patient functioning and caregiver burden.	Strong connections reduce burden, while negative relationship changes increase it, highlighting the role of mutuality in HF caregiver.
7	Gilotra et al. 2021 [33]	USA	Longitudinal	HF	N: 95	To examine caregiver HRQOL and burden changes over time, linked to patient and caregiver factors.	Patient HRQoL improves slightly over time, but caregiver HRQoL and burden remain stable; married caregivers correlate with better patient outcomes.
8	Grady et al. 2021 [34]	USA	Longitudinal (Comparative)	HT with and without MCS	N: 193 (n: 92 for candidates with MCS and *n* = 101 for candidates without MCS)	To compare HRQOL changes over time in caregivers of heart transplant patients with MCS versus without MCS	HT candidates with MCS show improved HRQoL, but their caregivers face higher burden despite high HRQoL.
9	Hayashi et al. 2021 [35]	Japan	Cross- Sectional	HF	N: 126	To characterize the role of and burden on caregivers of HF	HF caregivers spend extensive time on tasks impacting lifestyle, leading to physical and emotional burden from daily patient care.
10	Kanters et al. 2021 [36]	United Kingdom, Italy, Germany	Cross- Sectional	AF	N: 585	Study explores well-being, economic burden of providing informal care for AF	Caring for AF patients is burdensome but fulfilling, with societal costs from caregivers’ time and work absenteeism.
11	Lahoz et al. 2021 [37]	France, Germany, Italy, Spain, and United Kingdom	Cross-Sectional	HF	N: 361	To assess the burden among caregivers of patients with heart failure based on NYHA functional class	Caregivers of HF patients (LVEF ≤ 60%) experience increased anxiety, depression, and lower HRQoL, especially in severe cases.
12	Ghasemi et al. 2020 [38]	Iran	Cross-Sectional	HF	N: 140	To explore caregiver burden’s relationship to family functioning in caregivers of older adults with HF	Higher caregiver burden weakens family functioning in HF caregiving.
13	Subih et al. 2020 [39]	Jordan	Cross-Sectional	CVD	N: 200	To study on QoL in cardiac patients and caregiver burden	Healthcare providers should offer caregivers training and support to enhance coping, resilience, and care quality.
14	Szlenk-Czyczerska et al. 2020 [40]	Poland	Cross-Sectional	CVD	N: 161	To identify needs, emotional exhaustion, depersonalization, and personal accomplishment in home caregivers for CVD, along with related sociodemographic factors.	Caregiver burden is linked to advanced age, gender, marital status, unemployment, urban living, and high education; burnout prevention programs are recommended.
16	Durante et al. 2019 [41]	Italy	Cross-Sectional	HF	N: 505	To identify caregiver, patient predictors of burden in heart failure; assess if caregiver’s role in self-care raises burden	Caregiver and patient traits impact burden; caregiver involvement in self-care doesn’t heighten caregiver burden
17	Tavakoli et al. 2018 [42]	Iran	Qualitative	HF	N:14	To explain educational needs of family caregivers of HF patients	Themes: Care education, dignity, resilience for holistic healthcare improvement
18	Chi et al. 2018 [43]	USA	Mixed method	Hospice HF	N: 28	To explore caregiver challenges in advanced heart failure for home hospice	Patient care challenges, limited social support, communication barriers, and financial worries are prevalent themes
19	Bangerter et al. 2018 [44]	USA	Qualitative	HF	N: 16	To identify the most significant challenges facing HF caregivers	Balancing employment, lack of support, time, and caregiver health are key challenges.
20	Dirikkan et al. 2018 [45]	Turkey	Cross-Sectional	HF	N: 200	To assess the impact of caregiver burden on psychosocial adjustment in caregivers of HF patients under treatment.	Increased caregiver distress correlates with poorer psychosocial adjustment; lack of healthcare provider information adds burden.
21	Jackson et al. 2018 [46]	China	Cross-Sectional	HF	N: 458	To identify HF caregivers’ challenges, well-being and responsibilities	HF care affected caregivers’ emotions, social life, health, and had psychological, work, and economic implications
22	Sultan et al. 2018 [47]	Pakistan	Cross-Sectional	CVD	N: 257	To determine social support’s impact on caregivers’ burden and wellbeing in cardiovascular patient care	Social support mitigates caregiver burden’s impact on wellbeing, emphasizing emotional and tangible benefits.
23	van Wijnen et al. 2017 [48]	The Netherlands	Prospective cohort	Cardiac Arrest Survivors	N: 195	To assess caregivers’ well-being post-cardiac arrest, assessing function and factors influencing caregiver burden 12 months later	Strain is linked to emotional issues, perceived patient cognitive lapses, and lower QoL; burden persists for some at 12 months
24	Wingham et al. 2017 [49]	United Kingdom	Qualitative	HF	N: 22	To identify factors causing anguish, explore caregiver learning in demanding roles, adapting to challenges	Emotional impact, role definition, exclusion, and neglecting personal health emphasized
25	McIlfatrick et al. 2017 [50]	United Kingdom and Ireland	Mixed method (P1: Survey, P2: interview)	HF	(P1) N: 84 (P2) N: 30	To identify psychosocial factors in caregiver burden, assess support needs for advanced heart failure	Caregiver burden relates to depression, anxiety, and QoL scores. Seeking emotional support, desiring information, lack of support knowledge about the end-of-life expectations are key issues.
26	Hu et al. 2016 [51]	China	Cross-Sectional	HF	N: 226	To determine caregiver burden and its factors among family caregivers of HF patients	Financial strain, limited resources (social support, self-efficacy), and caregiver traits are factors linked to caregiver burden.
27	Sullivan et al. 2016 [52]	USA	Qualitative	HF	N: 63	To explore HF caregiving, uncover needs, and address concerns for a comprehensive understanding.	HF caregivers face competence, compassion, and self-care concerns, reflecting on their caregiving roles
28	Halm et al. 2016 [53]	USA	Qualitative	CABG	N: 32	To examine CABG caregivers’ burden and needs within 3 months post-surgery	Caregivers feel unprepared and need dedicated programs for guidance and resource access.
29	Vilchinsky et al. 2015 [54]	Israel	Cross- Sectional	ACS	N: 109	To test attachment’s moderation on burden-depression link post-ACS events over 12 months	Burden links to prolonged depression, worsened by attachment anxiety, impacting psychological distress.
30	Bozkurt Zinciret et al. 2014 [55]	Turkey	Cross-Sectional	HF	N: 138	To explore adult-child caregiver burden, factors, and depressive symptoms	Female caregivers with depressive symptoms face higher burden. caregiver and patient factors affect depressive symptoms and caregiving time correlates with burden severity, while education negatively correlates with burden.
31	Bahrami et al. 2014 a [56]	Iran	Qualitative	HF	N: 18	To study Iranian caregivers’ burden caring for HF patients	Lack of care knowledge, physical and psychosocial exhaustion, inadequate support
32	Bahrami et al. 2014 b [9]	Iran	Qualitative	HF	N: 19	To explore needs and issues of family caregivers for heart failure	Lack of care knowledge, info source inaccessibility, healthcare team guidance absence, and ambiguity coping due to disease’s unpredictable nature are key themes
33	Pressler et al. 2013 [57]	USA	Longitudinal	HF	N: 63	To assess changes in family caregivers’ time, task difficulty, control, depressive symptoms, anxiety, life changes, and HRQoL over 8 months.	Caregiving difficulty and time improve at 4 and 8 months; caregivers of severe symptom patients report higher anxiety and poorer health but experience some decrease in depressive symptoms and increased positive life changes.
34	Coleman et al. 2012 [58]	USA	Prospective	AF	N: 80	To examine caregiver, patient, thromboprophylaxis impact on AF burden interrelationship	Caregiver burden rises with schedule disruption, especially when caregiving exceeds 4 h weekly or patients are frail; financial strain relates to frequent follow-ups; female gender and time spent increase health-related burden.
35	Mochari-Greenberger et al. 2012 [16]	USA	Prospective	CVD	N: 423	To assess if caregiver burden link to lifestyle changes 1 year post family member’s cardiovascular hospitalization	Caregiver burden associates with worse diet and activity after discharge, impacting cardiovascular risk and highlighting need for intervention.
36	Hwang et al. 2011 [59]	USA	Cross-Sectional	HF	N: 76	To identify factors linking to caregiving and associated influences	Heart failure caregiving has varied family effects; post-hospital support is crucial to reduce negative impacts.
37	Ågren et al. 2010 [67]	Sweden	Cross-Sectional	HF	N: 135	To analyze HF caregiver burden levels and identify predictors	Poorer partner mental health, less control, and patients’ worse physical health predict higher caregiver burden focused on restricted freedom and social interaction.
38	Ågren et al. 2009 [60]	Sweden	Qualitative	HF after cardiac surgery	N: 13	To assess spousal needs post-cardiac surgery complications for HF patients	Security, rest, and inner strength are vital themes
39	Clark et al. 2008 [61]	United Kingdom	Qualitative	HF	N: 30	To analyze informal caregiving challenges in chronic HF	Informal caregivers provide emotional, physical, and advocacy support, managing both visible and subtle patient care fluctuations.
40	Saunders et al. 2008 [62]	USA	Cross-sectional	HF	N: 50	To understand caregiver burden factors in HF caregivers	Older age, more caregiving hours, caregiver health issues, depressive symptoms, and patient comorbidities predict higher burden; Caucasian ethnicity, relative caregivers, unemployment, and respite care correlate with increased burden
41	Luttik et al. 2007 [63]	The Netherlands	Cross-Sectional	HF	N: 357	To identify caregiver burden determinants in at-risk caregivers	Patients’ physical health correlates with caregiver burden via daily schedule disruption and physical strength loss; burden links mostly to partner mental health and personal care provision
42	Halm et al. 2006 [64]	USA	Cross-Sectional (comparative)	CABG	N: 166	To explore patient-spouse caregiver dynamics and role factors linked to post-CAB surgery burden	Female gender, poorer health, lower caregiver HRQOL, higher perceived gain, and caregiver competence relate to increased burden

*Abbreviations*: *ACS* Acute Coronary Syndrome, *AF* Atrial fibrillation, *CABG* Coronary Artery Bypass Graft, *CVD* Cardiovascular disease, *HCP* Health Care Provider, *HF* Heart Failure, *HRQOL* Health-Related Quality of Life, *HT* Heart Transplantation, *LVAD* Left Ventricular Assist Device Implantation, *LVEF* Left Ventricular Ejection Fraction, *MCS* Mechanical Circulatory Support, *NYHA* New York Heart Association, *OHCA* Out-of-Hospital Cardiac Arrest, *P* Phase, *STEMI* ST Elevation Myocardial Infarction,

### Data synthesis

Systematic reviews utilizing mixed methods offer an innovative strategy for tackling crucial inquiries within the realm of healthcare [65]. In the current investigation, we employed the updated JBI methodological guidance for conducting a mixed methods systematic review. Specifically, we utilized the convergent integrated approach for synthesizing data. The convergent integrated approach involves amalgamating data extracted from both quantitative and qualitative studies, incorporating a process known as data transformation. To achieve data transformation, we converted quantitative data into qualitative data through a method known as “qualitizing” which encompasses a narrative interpretation of the quantitative results. Subsequently, the textual descriptions, referred to as “qualitized data,” derived from the quantitative studies were aggregated and integrated with the qualitative data obtained from the qualitative studies.

For example, Dirikkan et al. [45] found that 84% of caregivers reported anxiety; this finding was qualitized as “most of the caregivers experience anxiety”. Then, this result was combined with qualitative themes from Bahrami et al. [56] where caregivers described ‘high levels of anxiety’ to form the integrated theme " Mental health problems”.

Data qualitization was conducted independently by two reviewers (F.B. and Z.M.) following JBI guidance. The categories and factors were developed by the authors through an iterative thematic analysis process. For this, study-level factors were first extracted (mapping), then reviewed inductively through thematic coding across qualitized and qualitative data to generate higher-order categories based on conceptual similarity (e.g., demographic factors from multiple studies were grouped under “individual characteristics”) [66]. To ensure rigor and trustworthiness, an audit trail was maintained, and iterative discussions were held to refine category interpretations. Discrepancies were resolved through consensus, with consultation from a third reviewer (M.N.) when necessary. Reflexive discussions among the review team were used to minimize interpretive bias.

**Fig. 2:**
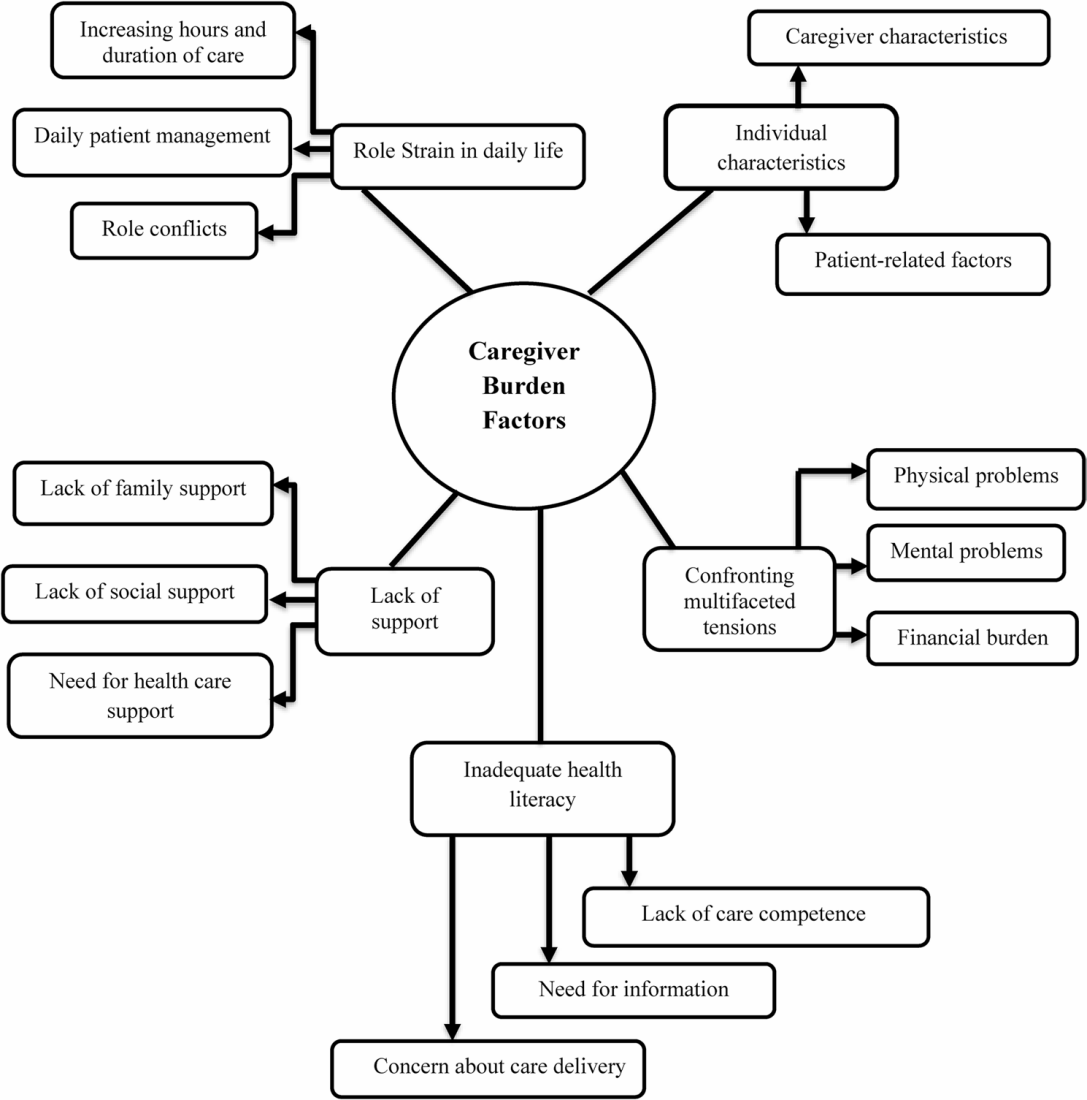
Conceptual framework regarding caregiver burden factors

The conceptual framework (Fig. 2) was developed through thematic analysis of the integrated qualitized and qualitative data, guided by JBI methodological recommendations, by synthesizing the identified categories and sub-categories into a visual model, derived from integrated data patterns across studies. The synthesis was led by F.B. and Z.M., with input from all authors during multiple review rounds.

Five categories consisting of 14 sub-categories constitute the factors contributing to burden in informal caregivers of CVD individuals.

## Results

### Study characteristics

In this systematic review, 42 articles were included, published between 2006 and 2023. The studies spanned various countries, with the USA (14), United Kingdom (6), Iran (4), Italy (4), Turkey (3), the Netherlands (3), Sweden (3), China (2), Denmark (2), Germany (2), Japan (1), Poland (1), Israel (1), Pakistan (1), Jordan (1), Spain (1), France (1), Slovenia (1), Ireland (1), and Canada (1) represented. Some studies were multicenter and conducted simultaneously across different countries.

Among the included studies, 21 articles employed a cross-sectional design [13, 30, 31, 35–41, 45–47, 51, 54, 55, 59, 62–64, 67], 8 were longitudinal [16, 29, 32–34, 48, 57, 58] and 13 studies utilized qualitative designs (phenomenology, content analysis, and mixed methods) [28, 42–44, 49, 50, 52, 53, 56, 60, 61, 68, 69] to explore the factors contributing to the caregiver burden of informal caregivers of cardiac patients.

Studies with higher methodological quality (with robust sampling and data analysis) were prioritized in the narrative synthesis, providing stronger evidence for key categories like “confronting multifaceted tensions.” Lower-quality studies (e.g., those with biases in measurement) were used supportively but with caution, ensuring the integration reflected overall evidence strength.

After data analysis, it emerged that 37 studies pertained to non-surgical cardiac patients (those with heart failure, acute coronary syndrome, myocardial infarction, cardiac arrest survivors, and arrhythmia), while 5 studies focused on cardiac surgery patients. One of the studies included in the analysis compared cardiac arrest survivors and myocardial infarction patients [31]. 

### Thematic analysis

The synthesis of data from the included studies unveiled five categories: individual characteristics (personal characteristics of the caregiver and patient-related factors), confronting multifaceted tensions, role strain in daily life, deficit in care knowledge, and lack of support (refer to Table 2).

**Table 2:** The summary of categories and factors related to caregiver burden in informal caregivers of cardiac patients

Categories	Caregiver burden factors
Individual characteristics	*Demographic characteristics of the caregiver*: Age, gender, education level, marital status and race *Patient-related factors*: - Physical (disease severity and comorbidities, patient readmission) - Financial (low income) - Psychological conditions (cognitive impairment) - Being single, old age, level of education.
Role Strain in daily life	- Role conflicts - Increasing hours and duration of care - Patient management for daily affairs
Confronting multifaceted tensions	- Physical problems: physical fatigue, ignoring health, unhealthy behaviors and poor physical habits - Mental health problems: depression, anxiety, fear, despair, loneliness - Financial burden: financial concerns and low income, unemployment, leaving work
Inadequate health literacy	- The need for information about the disease (prognosis and end-of-life care) - Lack of competence in providing proper care - Concern about serious errors in care delivery
Lack of support	- Lack of involvement of other family in care - Lack of social support: changes in social life, a reduction in interpersonal interactions, and disruption in family relationships - The need for health care support

#### Individual characteristics

##### Demographic characteristics of the caregiver

The findings of this systematic review study reveal that demographic characteristics can contribute to the emergence and augmentation of caregiver burden in informal caregivers of cardiac patients. Age, gender, education level, marital status, and race are among the factors involved.

The age of the caregiver is one of these factors. Several studies have shown a direct correlation between the age of the caregiver and the burden of care. The older the caregiver, the greater the caregiver burden to which they are exposed [38, 40, 41, 62]. However, only one study considered the young age of the caregiver as a factor for the caregiver burden [39].

Caregiver gender was another factor. In most studies, women experienced a higher burden while providing caregiving [38, 39, 41, 64], and caregiving in women was associated with higher levels of depression [55]. Only one study found a direct correlation between the caregiver burden and the male gender [58]. A low level of education among caregivers was another predictor of care strain [42, 43, 44, 54].

Other characteristics of caregivers include marital status. The results of the present study show that single caregivers were more vulnerable than married persons during the caregiving period and suffered more from depression [38] and a lower quality of life. It was also shown that patients with single caregivers were at a higher risk of developing complications than married ones [33]. Two studies also looked at ethnicity, and it was found that Caucasians endured more burden [62] and Blacks had a more positive view of caregiving [59]. This result likely reflects cultural, socioeconomic, or regional factors rather than inherent ethnic traits.

##### Patient-related factors

Physical, financial, and psychological conditions, along with the severity of the patient's illness, can contribute to caregiver burden. In this context, the patient's physical issues, such as memory impairment, confusion, and poor appetite [53, 63, 64], as well as factors like disease severity and comorbidities [11, 36, 37, 51, 57–59, 62], readmissions, and hospitalizations in medical centers, along with prolonged illness duration, have been linked to challenges in caregiving and disruption of caregivers' schedules [36, 51]. Patient characteristics, such as being single, having low income, and advanced age, have been demonstrated to be correlated with increased depression in caregivers [55]. Other influencing factors encompass cognitive impairment [31], poor mental health [11], and the and the patient's level of education [41, 51].

#### Role strain in daily life

Caring for chronic cardiac patients can bring new roles and responsibilities for caregivers. Role conflicts [49], increasing hours and duration of care [51, 61, 62, 70], patient management for day-to-day issues (medication management, meal preparation, patient transportation, and symptom monitoring) [37, 43, 46, 53, 57, 58, 61] have been associated with disruption of individual plans [57, 59] and a reduction in the caregiver's personal freedom [11, 44].

#### Confronting multifaceted tensions

The findings indicate that caregivers face various stressors while caring for patients with heart disease. These challenging tensions encompass physical, psychological, and financial aspects.

##### Physical problems

Caring for patients with heart disease had a negative impact on caregivers' quality of life, affecting both lifestyle and physical aspects. This impact manifested in physical fatigue [30, 35, 36, 43, 47, 57, 62, 63] and pain [46]. In this study, it was observed that certain caregivers tended to neglect their health and needs due to caregiving [44, 49, 52, 68], potentially leading to unhealthy behaviors and poor physical habits that may elevate the risk of cardiovascular disease [16]. The presence of chronic illness among informal caregivers could contribute to the burden associated with caregiving [38].

##### Mental health problems

Mental health problems in caregivers are significant factors interacting with caregiver burden, each potentially causing the other's existence. Study findings revealed that caregivers exhibited symptoms of depression [13, 37, 46, 55, 62], anxiety [37, 45, 46, 56, 57, 68], fear, despair, loneliness [28, 49, 56], and other emotional symptoms while attending to patients with heart disease [35, 54]. The psychological issues of caregivers may also contribute to an elevated burden [11, 50, 56, 63, 64].

##### Financial burden

The financial concern is another burden imposed by caregiving on informal caregivers. The results showed that financial worries and low income [30, 36, 38, 43, 45, 46, 51, 53, 55, 58], unemployment [39, 40, 62], difficulties in balancing work and caregiving, and leaving the job [44, 68] pose many challenges for caregivers.

#### Inadequate health literacy

The study found that caregivers lack adequate health literacy. The construct of health literacy includes three components: (1) knowledge of health, healthcare and health systems; (2) processing and using information in various formats in relation to health and healthcare; and (3) managing one’s own health through self-care and collaborating with healthcare professional [71].

Inadequate health literacy regarding the care of cardiovascular patients, a demand for information about the disease [38, 41, 42, 45, 49, 56, 57, 61], understanding the disease prognosis, end-of-life care [50] was seen. In addition, lack of competence in delivering appropriate care, and apprehensions about potential serious errors in care provision are noteworthy contributors to the burden and concerns experienced by informal caregivers [52].

#### Lack of support

The present study shows that the involvement of other family members in caregiving plays an important role in reducing the challenges faced by caregivers [38, 42, 58, 62]. Lack of social support, changes in social life, a reduction in interpersonal interactions, and disruption in family relationships are among the most significant and common causes of caregiver stress [11, 30, 42, 43, 45, 47, 51, 53, 56, 59, 68]. The need for healthcare support is one of the concerns of informal caregivers [45, 60].

## Discussion

This mixed-methods systematic review expands the existing literature by synthesizing evidence on caregiver burden across a broad spectrum of cardiovascular diseases. The present review integratively compiles evidence from acute, chronic, surgical, and non-surgical contexts of cardiovascular diseases. This approach enables the simultaneous identification of shared (transdiagnostic) mechanisms of caregiver burden and disease-specific manifestations, which are shaped by the disease trajectory, degree of unpredictability, and caregiving demands.

Informal caregivers, providing unpaid care to loved ones, contribute to the reduction of hospitalizations and medical costs, enhancement of cardiovascular and mental health, and decrease in mortality. Despite these myriad benefits, they face vulnerability to developing physical and psychological issues [70]. The present study aimed to assess the caregiver burden experienced by informal caregivers of patients with cardiovascular disease. This review included 42 studies from various geographical locations with distinct cultural, healthcare, and economic contexts. Many studies adopted cross-sectional designs, providing a snapshot of caregiver burden at specific points. Different cardiac conditions require varying levels of caregiving, resulting in distinct caregiver burdens for informal caregivers. The majority of patients in this review faced heart failure, possibly attributable to the intricate and chronic nature of heart failure management, requiring prolonged caregiving periods [11].

Caregiver burden is typically described as a complex and multifaceted concept encompassing physical, social, economic, psychological, and emotional stresses. In a more precise definition of caregiver burden, caregiver burden can be classified into two categories: subjective and objective [72].

Subjective burden refers to the attitudes and emotional reaction of the caregiver faced with the development of the care, such as a low emotional mood, anxiety, or depression. The objective burden arises from caregiver roles, impacting various aspects of the caregiver’s life such as work, social activities, and leisure [72].

In this systematic review, five distinct categories have been extrapolated from data synthesis to offer a comprehensive understanding of caregiver burden among informal caregivers. A closer look at the categories and sub-categories reveals that the current study’s findings encompass both the subjective and objective aspects of the caregiver burden and both dimensions together have led to a burden of care in the caregivers.

We discovered that individual characteristics, specifically the demographic traits of informal caregivers, play a crucial role in the development of burden in caregivers of patients with cardiac disease. Age serves as a predictor of caregiver burden, with older caregivers typically experiencing heightened care-related stress. The gradual advancement of age decreases physiological reserves, increases the risk of disease, and leads to a decline in individuals’ intrinsic capacities. Female informal caregivers of cardiac patients generally encounter higher burden, aligning with literature on CVD caregiving [73]. While male and female caregivers may exhibit distinct caregiving approaches, evidence suggests that their roles and relationships within the family should be taken into account, rather than exclusively concentrating on their genders [74]. Furthermore, individual informal caregivers and caregivers with lower educational levels are susceptible to higher levels of burden [39, 75]. These findings suggest the presence of a distinct group of caregivers vulnerable to caregiver burden in the CVD context, where acute events like cardiac arrests add unpredictability [28].

In our systematic review, we concentrated on caregiver and patient factors related to burden in caregivers of cardiac patients. The physical, financial, and psychological condition of cardiac patients directly influences the burden on their informal caregivers. Cardiac patients with severe conditions, more comorbidities, and frequent readmissions naturally require more effort from their caregivers, affecting their time, financial resources, and mental health. The severity of symptoms in cardiovascular disease and the disability associated with this condition are among the primary contributors to caregiver burden, as suggested by another study [76]. We also found that caring for chronic cardiac patients invariably reshapes the daily life of caregivers. The new roles and responsibilities can become overwhelming, especially when managing the patient’s daily needs becomes complex. This pushes them to adapt, sometimes straining their autonomy. Patients with cardiac disease are at risk for damaging a couple’s relationship, with changing spousal roles, the dissipation of a sense of partnership, worsening sexual problems, and caregiver burden [77, 78]. This review also revealed that informal caregivers are at risk of physical, psychological, and financial strains. This aligns with the findings of Bouchard et al.‘s review paper, indicating that nearly half of caregivers encountered at least one form of burden (physical, psychological, social, or emotional) while caring for patients with cardiovascular disease [70].

Physical challenges, such as fatigue, can pose potential health risks and lead to the neglect of caregivers’ own health. Existing research indicates an association between family caregiver strain and an elevated estimated risk for cardiovascular diseases, including conditions like hypertension and metabolic syndrome. This heightened risk extends to stroke, coronary heart disease, and overall mortality [16]. Therefore, it is essential to monitor informal caregivers for heart disease risks. The most prevalent mental health challenges for these caregivers are depression and anxiety. These psychological issues are commonly reported among informal caregivers of heart failure patients, especially when caring for patients at home without sufficient physical and emotional rest [79]. Financial problems exacerbate these challenges, underscoring the multifaceted nature of caregiver burden, especially where ongoing medical costs are high. Leaving work and low income could decrease caregivers’ quality of life and induce mental problems, which in turn increases their perceived burden [80].

Inadequate health literacy of caregivers is another contributing factor for caregiver burden. Inadequate knowledge about caring for cardiac patients can contribute to additional stress for informal caregivers. Similar associations between lack of knowledge and caregiver burden have been reported in other diseases [81–83]. Therefore, healthcare providers should conduct a comprehensive assessment of caregivers’ knowledge regarding patient care.

Informal caregivers lack social support and resources, and often have limited communication with other family members. So, they are susceptible to experience greater financial, physical and psychosocial costs and this can ultimately compromise the quality of care they are able to provide [18]. While seeking social support from social service and healthcare systems, informal caregivers often find their needs ignored by healthcare providers. They are frequently insufficiently informed about specific aspects of chronic diseases. Suboptimal communication with informal caregivers can result in caregiver distress and impaired competence in coping with uncertainty, potentially negatively affecting the patient’s well-being [11, 84]. Given that burden in informal caregivers of cardiac patients are associated with physical and psychological problems, interventions designed to cope with or reduce these burden are crucial [16].

Analyzing the factors affecting the caregiver burden, it can be said that these factors can be inter-related. For example, the lack of social and family support [85] or economic pressures due to loss of work and unemployment can lead to mental and psychological issues in the caregiver [86].

On the other hand, lower levels of education can be related to the low health literacy of caregivers and financial difficulties [87]. The caregiver burden is a very complex phenomenon, requiring several interrelated factors to develop.

Upon reviewing the literature, two systematic review studies with subjects relatively related to the current study were identified. Suksatan et al.‘s research focused on examining the caregiver burden and health outcomes in caregivers of HF patients revealed that an older age for both the caregiver and patient, the patient having a higher level of education, and the patient having a lower psychological quality of life were linked to an increased caring burden [25].

Another systematic review study was conducted to investigate the effects of care on caregivers of heart failure patients in the United States. This systematic review found that caregivers of HF patients face a significant burden, the wide variation in time spent caregiving, and the frequent experience of depression among caregivers, which may lead to increased healthcare resource use for caregivers [88].

Compared to previous systematic reviews, including those by Bjørnnes et al. (2019) and Suksatan et al. (2022), the present review goes beyond descriptions limited to a single specific disease by integrating findings across diverse cardiovascular disease diagnoses. While prior reviews have primarily documented caregiver challenges within narrowly defined clinical contexts, this synthesis demonstrates how similar burden domains (such as psychological distress and role strain) arise from different mechanisms depending on disease characteristics, thereby providing a more explanatory and transferable understanding of caregiver burden.

Out of the 42 articles included in the study, 12 articles have investigated the factors of caregiver burden in patients other than those with cardiac surgery and heart failure (such as arrhythmia, coronary artery disease, cardiac arrest, and heart transplantation, etc.). This synthesis demonstrates that caregiver burden manifests differently depending on the trajectory of cardiovascular diseases. In chronic and progressive conditions such as heart failure, caregiver burden typically has a cumulative and persistent nature, arising from long-term role strain, ongoing symptom management, and the gradual erosion of caregivers’ physical and psychological resources. In contrast, caregiving in acute or episodic conditions such as cardiac arrest or arrhythmias is associated with sudden onset, emotional shock, and high uncertainty, leading to an experience of intense but less predictable caregiver burden.

One of the key integrative insights from this review is the pivotal role of unpredictability in shaping caregiver burden. In many conditions beyond heart failure—including cardiac arrest, arrhythmias, and heart transplantation contexts—uncertainty regarding symptom recurrence, sudden deterioration of the patient’s condition, or treatment outcomes emerges as the dominant factor driving anxiety, emotional distress, and a sense of loss of control among caregivers. This mechanism is less prominent in chronic and predictable care trajectories, suggesting that uncertainty itself can function as a distinct pathway in generating caregiver burden.

Although the summarized caregiver burden domains in Table 2 may not appear innovative in isolation, their added value lies in the alignment and integration of quantitative and qualitative evidence across heterogeneous populations with cardiovascular diseases. This synthesis demonstrates how recurring burden domains emerge concurrently and interact with one another in different conditions, rather than presenting isolated and discrete findings from single-disease studies.

In this systematic review, the quality of the included studies was assessed using the MMAT, which is designed to evaluate studies without generating a composite numerical score. This approach allowed for a comprehensive evaluation of the studies’ methodological strengths and limitations, such as the appropriateness of study designs in capturing the complexities of caregiver burden, the rigor in data collection and analysis related to caregiver burden and coping, and the alignment between research questions and employed methods.

In the absence of a summary score, this evaluation supports cautious interpretation, recognizing that while some studies offer strong evidence regarding factors influencing caregiver experiences, others may present limitations that warrant careful consideration.

To address this critical issue, future research should prioritize the development and use of validated, standardized questionnaires. The application of uniform measurement tools would enhance comparability across studies, facilitate meaningful meta-analysis, and ultimately strengthen the evidence base.

### Strengths and limitations

This review is warranted as the first comprehensive mixed-methods synthesis of caregiver burden factors across diverse cardiovascular conditions, filling gaps left by prior condition-specific reviews and providing an integrated framework to inform broader, targeted support strategies for caregivers. In the present review, the included studies employed a variety of measurement instruments and questionnaires to assess the caregiver burden. This variation introduced substantial methodological heterogeneity, which significantly impeded our ability to perform a meta-analysis, which is often considered the gold standard for quantitatively synthesizing evidence. This limitation must be acknowledged as it impacts the interpreting the results and overall confidence in the conclusion drawn from this review.

Furthermore, this review included full-text, peer-reviewed, and English publications, potentially overlooking some evidence. Restricting inclusion to English-language publications, while practical, can lead to a “language bias” that disproportionately excludes studies from non-English-speaking regions, thereby underrepresenting diverse cultural perspectives and potentially overgeneralizing findings from English-dominant contexts. Date restrictions, such as limiting studies to those published between 2000 and 2023, help focus on contemporary evidence but can exclude historical data or emerging trends, leading to a temporally narrow view of caregiver burden. These limitations may reduce the comprehensiveness of our findings, particularly in capturing the full spectrum of cultural influences on informal caregiving.

The majority of studies were cross-sectional, offering a snapshot of caregiver burden without depicting its progression, particularly in chronic cardiac disease, where the burden level can be determined by the disease severity.

### Implication for practice

Healthcare providers must take a holistic view in order to understand the burden of providing informal care to people with cardiovascular diseases. They need to understand that informal caregivers must respond to care-recipients’ needs in all aspects of life. Therefore, it is advised to evaluate caregivers’ capacity to ensure that caregivers are properly supported in their endeavors.

Implementing routine assessments of caregiver burden using validated instruments is recommended, particularly for female, older, or less-educated caregivers, to quantify burden levels accurately [89]. Importantly, the identified differences in burden patterns suggest that caregiver assessments and interventions should be tailored to disease trajectory. Caregivers in chronic conditions may benefit from sustained support strategies targeting role overload and long-term coping, whereas those caring for patients with acute or unpredictable conditions may require early psychological support and crisis-oriented education.

From policy perspective, introducing financial support mechanisms, such as subsidies or tax relief for low-income caregivers, and expanding telehealth services to underserved regions can alleviate perceived caregiver burden and improve access to support resources. In addition, longitudinal studies are required to understand the temporal pattern of caregiver burden and its determinants. Additionally, randomized controlled trials evaluating psycho-educational interventions are needed to assess their efficacy in reducing caregiver burden.

## Conclusion

Caregiver burden is influenced by multiple interconnected factors including the caregiver’s individual demographics, patient-related challenges, and the increasing demands of daily caregiving roles. Caregivers often experience physical exhaustion, mental health problems, and financial difficulties, all of which contribute to their overall stress. Limited health literacy further complicates their ability to provide effective care and increases anxiety about making mistakes. Additionally, a lack of support from family, social networks, and healthcare systems leads to isolation and reduced resources. These challenges, while varying by cultural and socioeconomic context, underscore the need for targeted interventions to mitigate caregiver burden. Although the review findings highlight factors contributing to caregiver burden, but further research is needed to delve deeper into these areas.

## Supplementary Information

Supplementary Material 1.

## Data Availability

The datasets used and/or analyzed during the current study are available from the corresponding author on reasonable request.

## Abbreviations

CVD: Cardiovascular Diseases
DALY: Disability-Adjusted Life Years
GBD: Global Burden of Disease
HF: Heart Failure
JBI: Joanna Briggs Institute
MMAT: Mixed Methods Appraisal Tool
PRISMA: Preferred Reporting Items for Systematic Review and Meta-Analysis
PROSPERO: Prospective Register of Systematic Reviews
